# Connecting Metabolism to Mastitis: Hyperketonemia Impaired Mammary Gland Defenses During a *Streptococcus uberis* Challenge in Dairy Cattle

**DOI:** 10.3389/fimmu.2021.700278

**Published:** 2021-06-29

**Authors:** Turner H. Swartz, Barry J. Bradford, Laman K. Mamedova

**Affiliations:** ^1^ Department of Animal Science, Michigan State University, East Lansing, MI, United States; ^2^ Department of Animal Sciences and Industry, Kansas State University, Manhattan, KS, United States

**Keywords:** mastitis, ketosis, metabolism, inflammation, immune function

## Abstract

β-hydroxybutyrate (BHB) has been associated with disease incidence in early lactation dairy cattle, but such associations do not demonstrate causation. Therefore, the objective of this study was to examine the effects of BHB during an intramammary *Streptococcus uberis* challenge. A secondary objective was to elucidate the mechanisms behind BHB effects on cytokine transcript abundance using the RAW 264.7 cell line. Late lactation multiparous dairy cows (n = 12) were continuously infused intravenously with either BHB to induce hyperketonemia (target concentration: 1.8 mM) or with saline (CON) for 72 h during a *S. uberis* intramammary challenge. Body temperature, dry matter intake (DMI), milk production, and milk *S. uberis* cfu were measured daily until one week post-challenge. Blood samples were collected during infusion to assess changes in metabolism (glucose, insulin, glucagon, NEFA, and cortisol) and systemic inflammation (IL-1β and SAA). Mammary biopsies were conducted at 72 h post-challenge to assess transcript abundance of inflammation-associated genes. BHB-infused cows exhibited a delayed febrile response, noted by a lesser vaginal temperature during the final day of infusion, followed by a greater vaginal temperature 6 d post-challenge. Consequently, BHB-infused cows had greater *S. uberis* cfu on d 4, 6, and 7 as compared to CON. Accordingly, BHB-infused cows consumed less DM, produced less milk, had reduced blood glucose, and had increased cortisol concentrations, however, no effects were seen on other systemic parameters or transcript abundance of inflammation-related genes in mammary tissue. To elucidate mechanisms behind the impaired immune defenses, RAW 264.7 cells were transfected with a GPR109A siRNA for 24 h and then treated with or without 1.8 mM BHB and challenged or left unchallenged with *S. uberis* for an additional 3 h. Transfection with siRNA reduced *Gpr109a* by 75%. Although BHB treatment did not significantly increase *Il10*, GPR109A knockdown as compared to the scrambled control reduced *Il10* by 90% in *S. uberis* challenged macrophages treated with BHB, suggesting that macrophage immune responses to *S. uberis* can be altered *via* a GPR109A-dependent mechanism. Taken together, these data suggest that BHB altered the immune response promoting tolerance toward *S. uberis* rather than resistance.

## Introduction

The postpartum period is a critical time in a dairy cow’s life when the prevalence of disease is greater than any other period during adulthood. Most peripartum cows experience negative energy balance due to increased energy demand required for lactation in addition to reductions in feed intake. Consequently, dairy cattle mobilize fat reserves for the purpose of ATP production, however, not all fatty acids are completely oxidized, resulting in the production of β-hydroxybutyrate (BHB), a major ketone body. Hyperketonemia is defined as an abnormal increase in circulating ketone bodies that is a sign of the health disorders more commonly known as clinical or subclinical ketosis. While the incidence of subclinical ketosis is variable between dairy herds (1), this disorder is generally quite common with some large field studies reporting nearly half of all postpartum cows having at least one BHB test ≥ 1.2 mM (2, 3). Clinical and subclinical ketosis have been associated with increased incidence of mastitis in early lactation dairy cattle (4, 5). Indeed, the incidence of clinical mastitis, typically caused by bacterial infection, is greater during the first few weeks of lactation (6, 7), suggesting that negative energy balance may predispose the dairy cow to infectious disease agents. As such, we sought to elucidate the direct role of BHB on mammary gland immune defenses.

*In vitro* models have consistently found that BHB impairs immune function, in particular BHB treatment reduced neutrophil bacterial killing abilities (8), impaired lymphocyte function (9, 10), and reduced phagocytosis in bovine milk macrophages (11). The anti-inflammatory properties of this metabolite are well-accepted now, even to the degree that increasing BHB concentrations through diet manipulations has been proposed as a therapy for human inflammatory disorders such as epilepsy, gout, Alzheimer’s, inflammatory bowel disease, and type 2 diabetes (12). With that said, little research has examined BHB-mediated effects during live infectious disease challenges using *in vivo* methods. Moreover, the mechanism behind BHB effects on immune function have not been fully investigated during infectious disease challenges, but past research has suggested that this effect may be mediated through the G-protein-coupled receptor GPR109A [also known as HCAR2; (13)].

*Streptococcus uberis* is a common environmental mastitis pathogen accounting for approximately 15 to 20 percent of all mastitis cases (14), and is responsible for a large proportion of clinical and subclinical mastitis cases occurring during the dry period and early lactation (15). As such, hyperketonemia may be a risk factor for *S. uberis* infections as environmental streptococci have a greater intramammary infection rate during the first month of lactation (16). Therefore, the objective of this experiment was to examine the effect of BHB on immune responses and metabolic parameters during an intramammary *S. uberis* challenge. Because macrophages are critical immune cells for the clearance of *S. uberis* (17), a secondary objective was to determine the role of GPR109A on BHB-mediated signals in *S. uberis* challenged macrophages. We hypothesized that BHB would impair immune responses resulting in a more severe intramammary infection.

## Materials and Methods

The animal experiment was conducted at the Kansas State University Large Animal Research Center in Manhattan, KS. All experimental procedures involving animals were approved by the Kansas State University Institutional Animal Care and Use Committee (Protocol 4236).

### Cow Selection, Housing, and Feeding

Late lactation (mean ± SD; 354 ± 51 DIM), multiparous (3.4 ± 1.7 parity) Holstein cows (n = 12) were purchased from a local dairy farm. Late lactation cows were chosen to avoid the confounding effects of the numerous physiological changes occurring in early lactation dairy cattle. All cows were clinically healthy at the time of purchase and had no clinical history of mastitis. Approximately 14 d before the experimental period (just prior to purchase), individual quarter milk samples were collected aseptically to screen for pre-existing infections using methods outlined by the National Mastitis Council (18). Only cows that were culture-negative for all four quarters were purchased. One week prior to the experimental period, cows were hauled from the local dairy farm to Kansas State University. Upon arrival, cows were weighed (759 ± 89 kg) and a body condition score (scale, 1-5; 3.8 ± 0.4) was recorded. As stated previously, cows arrived at the university one week prior to the experimental period, which was done to provide an adaptation period before administering treatments. During this time, individual quarter milk samples were collected again 3 d before challenge and just prior to the challenge (d 0) to verify that all four quarters of the mammary gland in every cow remained culture-negative. Moreover, individual quarter foremilk samples were collected for somatic cell count (SCC) quantification prior to challenge and all quarters to be challenged had a SCC < 250,000 cells/mL.

Cows were housed at the Large Animal Research Center in an animal room with individual pens fitted with a waterer, feed bunk, and rubber mats bedded with shavings. This animal facility is temperature controlled, which was set at 21°C. Cows were milked twice daily at 530 and 1730 h using a portable milker, which was cleaned after each milking. All cows were fed twice daily (530 and 1730 h) of the same diet balanced to meet the requirements of a late lactation cow producing 34 kg/d of milk consuming 24 kg/d of dry matter. The diet consisted of alfalfa and grass hay, SweetBran (Cargill Corn Milling, Blair, NE), soybean hulls, a custom-made grain pellet, a mineral mix, and molasses.

### Treatments

Cows were blocked by parity (2 or 3+) and milk yield, and randomly assigned within block to either receive an intravenous infusion of 2.5 M **BHB** sodium salt (Ketotech, Inc., Seymour, IL; n = 6) or a 2.5 M NaCl solution (**CON**; Fisher Scientific, Pittsburgh, PA; n = 6). As discussed later, BHB cows were infused at a rate to maintain a 1.8 mM BHB concentration in blood, and the infusion rate for the CON cow was matched with the infusion rate for the BHB cow within each block. The BHB infusate was prepared similarly as was done before (19). To achieve a 2.5 M BHB solution, 315.2 g of BHB sodium salt was dissolved in distilled, deionized water for a total volume of 1.0 L, followed by mixing. The BHB solution was then centrifuged for 1 h, filtered, pH adjusted to 7.4, sterilized in an autoclave, and stored at 4°C in a sterile 20 L Nalgene jug. A 2.5 M saline solution was prepared for CON cows by dissolving 146.1 g of sodium chloride into distilled, deionized water for a total volume of 1 L. The saline solution was also pH adjusted to 7.4, sterilized in an autoclave, and stored at 4°C in a sterile 20 L Nalgene jug.

### Catheterization, Infusion, and Blood Sampling

One day prior to administering treatments, cows were fitted with contralateral jugular catheters. One catheter was used for continuous infusions of the treatments, whereas the other catheter was used for blood sampling. Cows were restrained inside a chute using a halter. After clipping the hair around the jugular vein, the area was washed with alternating betadine surgical scrub and 70% ethanol once, then a 2-3 mL lidocaine bleb was administered, followed by the alternating scrub for at least two more times. Using one hand with sterile gloves, the jugular vein was occluded and the vein was localized with a 14 G and 6.25 cm introducer (MILA International, Erlanger, KY) using a percutaneous technique. Once the introducer was in the vein, gas-sterilized Tygon tubing (Saint-Gobain Performance Plastics, Akron, OH) was passed through the introducer (up to 25 cm). After removing the introducer, the exteriorized tubing was sutured to the skin using a tape butterfly. A tubing adapter (18 G, BD, Franklin Lakes, NJ) was fitted to the external end of the tubing, then flushed with approximately 5 mL sterile heparinized saline (20 IU/ml; Fisher Scientific) and fitted with a Luer-Lok™ cap (BD; Franklin Lakes, NJ). The procedure was repeated with the contralateral jugular vein. Catheters were wrapped and secured using Elastikon tape around the neck of the animal and an abdominal wrap was then placed around the Elastikon tape.

To continuously infuse the treatments into each cow, a gas-sterilized infusion line (Saint-Gobain Performance Plastics) was placed inside the Nalgene jug containing the treatments. This infusion line was then threaded through a small hole in the lid of the Nalgene jug, and fitted through a peristaltic pump (Baxter Flo-Gard 6201, Baxter Healthcare, Deerfield, IL), and finally attached to the catheter. During the infusion, cows were tethered using a halter but had access to feed and water and the ability to stand up and lie down. Blood samples were collected every hour from the contralateral catheter to monitor BHB concentrations and adjust the infusion rate on the pump to maintain a target concentration of 1.8 mM BHB in the treatment group. Within each block, the infusion rate was matched between cows. In other words, if the infusion rate for the BHB cow was increased, then the infusion rate for the CON was also equally increased, and vice versa. This was done to ensure that the sodium and total osmolyte infusion rate was the same between treatment groups.

Hourly ~1 mL blood samples were taken to monitor BHB concentrations, which was done by placing a small drop of blood on a BHB strip inserted inside a handheld meter (Precision Xtra, Abbott Laboratories, Abbott Park, IL). In addition to the hourly blood samples, ~10 mL blood samples were collected at 0 (just prior to challenge), 6, 12, 18, 24, 36, 48, 60, and 72 h post-challenge. Blood was dispensed into an EDTA tube, placed on ice, and then upon return to the lab, plasma was separated using centrifugation and stored at -80°C. For blood sampling, catheters were flushed with sodium citrate (sterile 3.5% solution; ~3 mL; Fisher Scientific) and then approximately 5 mL of blood was collected and discarded. After that, the blood sample was collected, and the catheter was flushed again with sodium citrate and then re-capped.

### Plasma Analyses

Plasma was analyzed for glucose (Wako Chemicals, Richmond, VA) and NEFA (Wako Chemicals) concentrations using enzymatic colorimetric procedures. Insulin (Mercodia, Uppsala, Sweden), glucagon (Mercodia), cortisol (G-Biosciences, St. Louis, MO), serum amyloid A (SAA; Tridelta Development, Ltd., Kildare, Ireland), and IL-1β (ThermoFisher Scientific, Waltham, MA) were quantified using bovine-specific ELISA. The insulin and SAA kits are frequently used in bovine research and were validated with acceptable performance by the manufacturer, however, glucagon, cortisol, and IL-1β assays required internal validation. As such, spike and recovery assessment for each ELISA was conducted to evaluate assay performance. The glucagon and cortisol assays had spike and recovery percentages of 103% and 94% respectively, and therefore these assays were considered valid. The IL-1β ELISA, however, had poor recovery (69%) when using the assay diluent provided with the kit. As such, modifications were made to the IL-1β ELISA protocol to improve assay performance. Spike-in recovery assessments were done using TRIS buffer (Fisher Scientific) as the assay diluent. A 1:2 plasma dilution yielded poor recovery (62%), however, a 1:4 plasma dilution improved assay performance (88% recovery). As such, all samples were diluted in TRIS buffer using a 1:4 dilution, and standards were also diluted using the same diluent. All other procedures were conducted using the guidelines provided by the manufacturer. Inter-assay and intra-assay coefficient of variations were 7.7 and 6.3%, 1.9 and 6.6%, 5.0 and 4.7%, 8.9 and 7.8%, 12.9 and 13.0%, 1.0 and 5.2%, and 8.0 and 7.8% for glucose, NEFA, insulin, glucagon, cortisol, SAA, and IL-1β, respectively.

### Intramammary Challenge

The intramammary challenge strain was *S. uberis* 0140J (provided in kind by Dr. John Lippolis, USDA-ARS), a well-characterized strain originally isolated from a naturally occurring intramammary infection (20). Procedures were conducted similarly to Kester et al. (21). A frozen stock culture in 10% skim milk kept at -80°C was streaked for isolation on esculin blood agar and incubated at 37°C overnight. Two colonies were transferred into 25 mL of Todd-Hewitt broth and grown at 37°C on an orbital shaker (200 rpm) in an incubator at 37°C for 7 h to reach stationary phase. The broth culture was centrifuged at 2,500 × *g* for 20 min at 4°C, and the pellet resuspended in 20 mL of sterile PBS (Dulbecco’s PBS Ca^2+^ and Mg^2+^ free; ThermoFisher Scientific). The broth culture was serially diluted in sterile PBS to achieve approximately 1 × 10^4^ cfu/mL for the challenge inoculum. The concentration was determined by drop-plating 10 µL of a 1:10 dilution of the challenge inoculum in quadruplicate on esculin blood agar and counting colonies after incubation at 37°C for 24 h. Before infusion, teats were cleaned with cotton balls soaked in 70% ethanol. The challenge dose (1 mL in each quarter) was administered in the right rear and right front quarter *via* a teat cannula (Jorgensen Laboratories Inc., Loveland, CO) immediately following milking. The infusion was dispensed through the teat canal and massaged upward to aid in the dispersion of the *S. uberis* into the mammary gland. Upon completion of the study, all cows were slaughtered, and therefore, no antibiotics (intramammary or systemic) or any supportive therapies (such as a NSAID, etc.) were provided following the challenge.

### Bacterial Enumeration and SCS

Bacterial enumeration was conducted from milk collected from the right front challenged quarter daily for 7 d post-challenge. Eight serial 10-fold dilutions were made in a sterile 48-well plate in duplicate using sterile PBS as the diluent. For each dilution and duplicate, four 10 µL replicates were dropped on the surface of 0.1% esculin Trypticase soy agar (TSA) and following 24 h incubation at 37°C, the colonies were counted at the appropriate dilution and averaged. For the lower limit of detection, pour plates were used where 1 mL and 0.1 mL of undiluted milk were dispensed in 12 mL of liquid 0.1% esculin TSA in duplicate, and colonies were counted following incubation at 37°C for 24 h. Bacterial counts were expressed as the number of colony-forming units per milliliter of milk.

Foremilk samples were collected from the right front quarter daily starting just prior to challenge (d 0) until 7 d post-challenge. Samples were refrigerated and within four days of collection sent to a DHI laboratory (MQT labs, Kansas City, MO) for SCC quantification using a cell counter (Bentley FCM, Bentley Instruments, Chaska, MN). Milk samples that were grossly clinically infected were strained with a cheese cloth to remove clots and diluted 1:10 in PBS before being sent to the laboratory, and SCC was calculated using the dilution factor. Quarter SCC was transformed to somatic cell score (SCS = log_2_(SCC/100,000) + 3).

### Mammary Biopsies

Biopsies (~200 to 600 mg of tissue) was taken from the left rear (unchallenged) and right rear (challenged) quarters 72 h after *S. uberis* intramammary challenge. Biopsy procedures were done similarly to Daley et al. (22). In brief, cows were sedated with IV administration of xylazine (0.01-0.05 mg/kg of body weight) into the coccygeal vessel. The biopsy site was located at the upper part of the rear udder (if the udder was divided into thirds, the biopsy site was located approximately one-third down from the top of the rear udder). The biopsy site was clipped and cleaned using alternating betadine surgical scrub and 70% ethanol once, followed by subcutaneous administration of a local anesthetic (6 mL of 2% lidocaine) just above the incision site, and then two more rounds of alternating surgical scrubs. Using aseptic technique, a 2 to 3 cm vertical incision was made using a number 10 scalpel. The biopsy tool [described by Farr et al. (23) and Daley et al. (22)] was attached to a cordless drill and inserted into the mammary tissue approximately 7.5 cm deep. A core sample was taken using clockwise drill action. Tweezers were used to remove the core sample from the biopsy tool. The sample was immediately blotted on a petri dish to remove blood and snap frozen in liquid nitrogen. Samples were kept on dry ice until biopsies were completed and then stored at -80°C. Immediately after the procedure, pressure on the incision site was applied for approximately 20 min using a sterile towel to achieve hemostasis. Five to eight stainless steel surgical staples were used to oppose the skin incision, an aluminum bandage was applied, and staples were removed 7 d post-biopsy.

### RNA Isolation and RT-PCR

Mammary tissue was placed in TRIzol™ (ThermoFisher Scientific), kept on ice, and then homogenized. Total RNA was isolated using the RNeasy Lipid Tissue kit (Qiagen, Germantown, MD). RNA was quantified using spectroscopy (Take3, Biotek Instruments, Inc., Winooski, Vermont) and purity was assessed using the 260/280 ratio (2.1 ± 0.1). RNA integrity (RIN) was assessed using an Agilent Bioanalyzer 2100 (Agilent Technologies, Santa Clara, CA). All samples had inferior RNA quality (3.4 ± 1.2). Additional attempts were made to re-isolate RNA from frozen tissue samples; however, these efforts were not successful in improving RNA quality. Treatment did not influence RNA integrity (left rear quarter, *P* = 0.78; right rear quarter, *P* = 0.34). As such, we proceeded with cDNA synthesis and real-time PCR, although we acknowledge the shortcomings of using RNA samples with substantial degradation. Total RNA (2 μg) was used as template for the reverse transcriptase reaction using random primers (Bio-Rad Lab. Inc., Hercules, CA). Quantitative real-time PCR was performed (QuantStudio 6 Flex Real-Time PCR System, Applied Biosystems, Foster City, CA) in duplicate with 200 nM gene-specific forward and reverse primers with iTaq Universal SYBR Green Supermix (Bio-Rad Lab. Inc., Hercules, CA). Primers were designed from bovine GenBank sequences and were designed to amplify an intron-spanning region of the gene and validated by identifying a single amplicon from the melt curve analysis. Primer sequences, accession numbers, and efficiencies are provided in **Table 1**. Primer efficiency was calculated using a 6-point curve. Transcript abundance was quantified using the relative expression ratio from Pfaffl (24) with the geometric mean of *RPS9* and *UXT* used to normalize values.

**Table 1:** Sequence, accession number, and primer efficiency for analyzed transcripts.

Gene^1^	Sequence	Accession number	Efficiency
*RPS9*	F: GAACAAACGTGAGGTCTGGAGG R: TTACCTTCGAACAGACGCCG	DT860044.1	0.90
*UXT*	F: ATTGAGCGACTCCAGGAAGC R: GGGACCACTGTGTCAACGAA	NM_001037471.2	0.90
*IL1β*	F: TAGCCCATGTGTGCTGAAGG R: TGCAGAACACCACTTCTCGG	NM_174093.1	1.10
*IL8*	F: GTCTGCCTAAACCCCAAGGA R: TTGCTTCTCAGCTCTCTTCAC	NM_173925.2	1.18
*IL10*	F: GGCGCTGTCATCGCTTTCT R: TGGCTTTGTAGACACCCCTC	NM_174088.1	0.91
*TNFA*	F: AGAGGGAAGAGCAGTCCCCA R: GGGCTACCGGCTTGTTACTT	NM_173966.3	0.85
*CCL5*	F: CCATGGCAGCAGTTGTCTTTA R: ACTTCTTCTCTGGGTTGGCG	NM_175827.2	0.96
*LTF*	F: CGGACAAGTGGGAAAGAAAGA R: TGGTTCCTCTCAAGTTCCCCA	XM_015459655.2	0.83
*NLRP3*	F: CTTTCTGGACTCTGACCGGG R: CTCCCATTCTGGCTCTTCCC	NC_037337.1	1.30
*GPR109A*	F: GCCGTGGATAGGTACTTCCG R: ATGGTGATGCCCCACAAGAG	XM_015475510.1	0.96

^1^RPS9, Ribosomal Protein S9; UXT, Ubiquitously Expressed Prefoldin Like Chaperone; IL, interleukin; TNFA, tumor necrosis factor α; CCL5, chemokine ligand 5; LTF, lactoferrin; NLRP3, NLR family pyrin domain containing 3; GPR109A, G-protein coupled receptor 109A.

### Clinical Outcomes

Milk yield was weighed at each milking using an electronic scale and summed by day. As-fed feed intake was recorded daily by weighing refusals and subtracting it from feed offered and adjusted by the dry matter content of the diet to determine daily dry matter intake (DMI). Vaginal temperature was recorded hourly using a data logger (iButton DS1922L, Thermochron, Baulkham Hills, NSW, Australia) attached to a blank CIDR (provided in kind by Zoetis Inc., Parsippany-Troy Hills, NJ). The data logger was inserted just prior to challenge and temperatures were averaged by day for 7 total days. For these variables, d 0 represented the first 24 h post-challenge, d 1 represented 25 to 48 h post-challenge, and so on. For milk yield and DMI, daily data recorded for 3 d prior to challenge was averaged, reported as baseline data in the figures, and was used as a covariate in their respective statistical models. Clinical signs of mastitis were recorded at each milking by the same observer. This included changes in milk appearance including clots, watery consistency, or discoloration, in addition to signs of inflammation such as swelling or redness of the quarter. To assess whether treatment influenced onset or time to onset of clinical signs, only data from the right front challenged quarter was used, as to avoid any influence that the mammary biopsy may have had on clinical signs of mastitis in the right rear challenged quarter.

### RAW 264.7 Cell Culture Experiment

To complement the results of the *in vivo* study and to elucidate some mechanisms, we assessed the effects of BHB, *S. uberis* challenge, and GPR109A silencing using a 2 × 2 × 2 factorial design (8 treatment groups with 9 replicates per group) on mouse macrophages (RAW 264.7 line). These cells were cultured in DMEM supplemented with 1% L-glutamine and 10% heat-inactivated fetal bovine serum. Twenty-four-well plates were seeded with 1 × 10^6^ cells per well and incubated in a humidified atmosphere overnight at 37°C and 5% CO_2_. After this, cells were transfected with 25 nM GPR109A siRNA (ON-TARGETplus SMARTpool, Dharmacon, Horizon Discovery Group Company, Cambridge, England) or nontargeted control (Mission siRNA Universal Negative Control #1, cat. no. SIC001, Sigma Aldrich) using TransIT-TKO Transfection Reagent (Mirus Bio LLC, Madison, WI) for 24 h. The transfection procedure was based off of a previous study (25) and a preliminary experiment conducted in triplicate in our lab which found 85% *Gpr109a* knock-down. Media was then removed and fresh media with or without 1.8 mM BHB (Sigma Aldrich, St. Louis, MO) and with or without 2 × 10^7^ cfu *S. uberis* (0140J strain) were added for 3 h.

For transcript abundance, cells were washed once with cold PBS, TRIzol_™_ (ThermoFisher Scientific) was added, and samples were stored at -80°C. The following day total RNA (2 μg) was isolated (Zymo Research, Irvine, CA) following manufacturer’s recommendations. RNA was quantified using spectroscopy and purity was assessed using the 260/280 ratio (mean ± SD, 2.01 ± 0.04) and then stored at -80°C. RNA integrity (RIN) was assessed on random subset of samples (n = 22; mean ± SD, 8.5 ± 2.1) using Agilent Bioanalyzer 2100 (Agilent Technologies). Complementary DNA was synthesized the day following RNA isolation. Total RNA was used as template for the reverse transcriptase reaction using random primers (Bio-Rad Lab. Inc., Hercules, CA). Quantitative real-time PCR was performed (QuantStudio 6 Flex Real-Time PCR System, Applied Biosystems) in duplicate with 200 nM gene-specific forward and reverse primers with iTaq Universal SYBR Green Supermix (Bio-Rad Lab. Inc.). Primer sequences and efficiencies are reported in our previous work (26). Transcript abundance was quantified using the relative expression ratio from Pfaffl (24) and normalized to *B2M*.

To determine if transfection or BHB treatment impacted cell viability, we assessed resazurin metabolism. Cells were transfected for 24 h and then treated with 1.8 mM BHB or not following the same treatment protocol as previously mentioned in black-walled, clear bottom 96-well plates (n = 8 wells per treatment group). Following incubation with treatments, media was removed and fresh media with 20 µL of a 0.15 mg/mL solution of resazurin sodium salt (Sigma-Aldrich) was added directly to RAW cells in 200 µL of media. Cellular metabolism was assessed by measuring fluorescent resorufin formation in relative fluorescent units (RFU) after 4 h of incubation with excitation at 530 nm and 590 nm emission using a fluorometric plate reader (Synergy HTX; BioTek Instruments Inc., Winooski, VT) and Gen5 software (BioTek Instruments Inc.). Results are expressed as a percentage of the control.

### Statistical Analyses

Linear mixed models were conducted using PROC GLIMMIX (SAS 9.4, SAS Inst., Cary, NC). For DMI, milk yield, plasma and milk analyses, the model included the fixed effects of treatment, time (repeated measure), and the two-way interaction with time, with the random effects of cow and block. The first order autoregressive covariance structure was used unless the outcome variable had uneven sampling intervals, in which case spatial power was used instead. When available, data collected prior to applying treatment were tested as a covariate to account for individual differences between cows. For mRNA abundance, each quarter was analyzed separately, and the model included the fixed effects of treatment with the random effects of cow and block. Backwards elimination was used to remove non-significant terms until all variables in the model had a *P* < 0.20. Treatment, time (when applicable), and their interaction were forced into the model regardless of significance. Finally, a Cox proportional hazards model (PROC PHREG) was used to assess treatment effects on the onset or time to onset of clinical signs of mastitis following challenge.

For the RAW 264.7 mouse macrophage experiment, data were analyzed as a 2 × 2 × 2 factorial using PROC GLIMMIX (SAS 9.4). The model included the fixed effects of BHB, *S. uberis* challenge, and GPR109A silencing as well as all two- and three-way interactions and the random effect of cell culture plate. Two orthogonal contrasts (LSMESTIMATE statement with Bonferroni adjustment) were conducted to assess BHB effects during a *S. uberis* challenge (BHB + *S. uberis versus S. uberis*) and to assess BHB effects during a *S. uberis* challenge when GPR109A was knocked down (BHB + *S. uberis versus* GPR109A siRNA + BHB + *S. uberis*).

In all models, residuals were assessed for normality and outliers (PROC UNIVARIATE). If an outcome variable was non-normally distributed, the natural logarithmic transformation was used. Significance was declared at *P* ≤ 0.05 and trends at 0.05 < *P* ≤ 0.10.

## Results

### *In Vivo* Experiment

**Figure 1:**
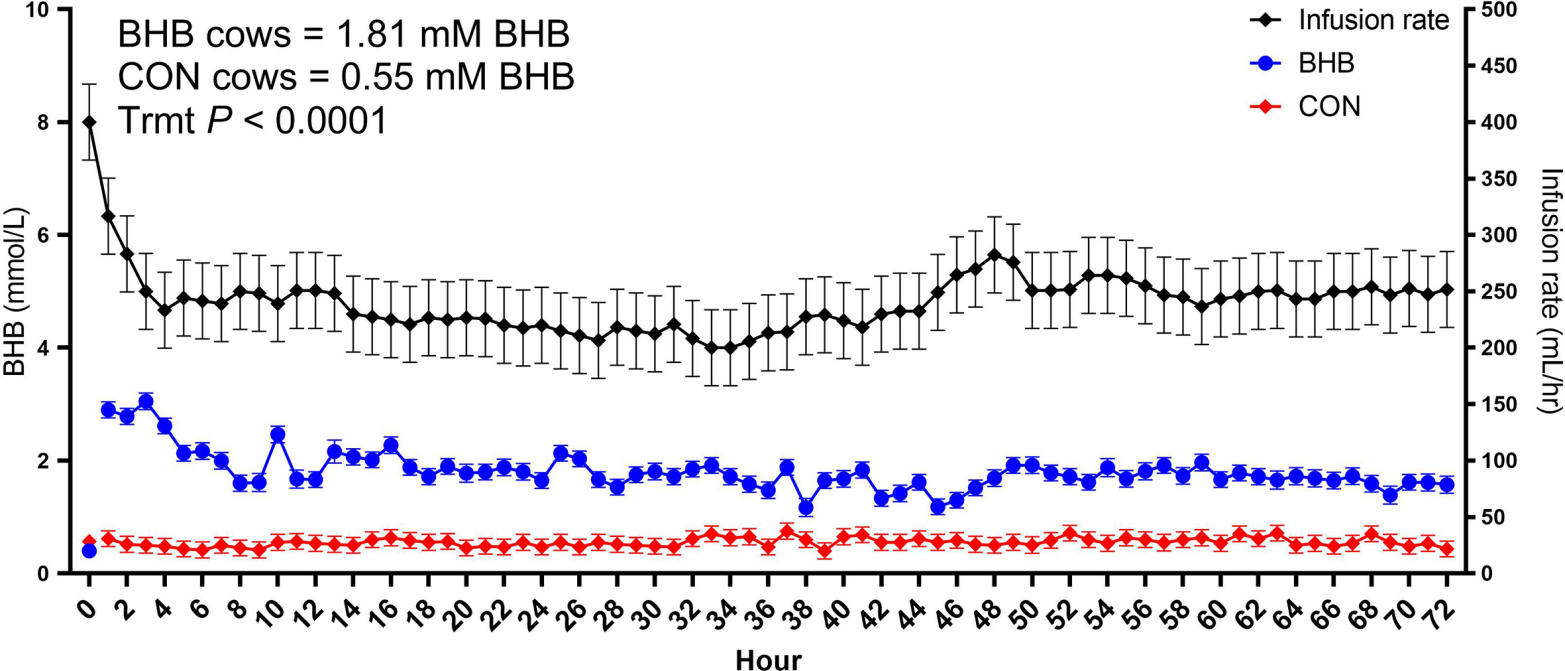
Intravenous infusion of sodium β-hydroxybutyrate (BHB) induced hyperketonemia. Late lactation multiparous cows (n = 12) were blocked by parity and milk yield and randomly assigned to either receive a 72 h intravenous infusion of 2.5 M Na-BHB or 2.5 M NaCl (CON). Blood BHB concentrations were monitored on an hourly basis, and the infusion rate was adjusted accordingly to maintain a target concentration of 1.8 mM BHB in the BHB-infused cows. Infusion rates were matched between CON and BHB cows to equilibrate sodium concentrations of the infusate between cows. All cows were challenged with *S. uberis* in the right rear and right front quarters of the mammary gland at the start of the infusion. Data are presented as LSM ± SE.

**Figure 2:**
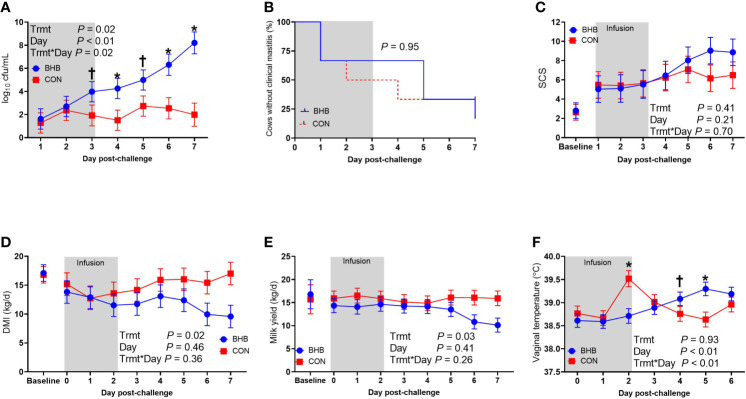
Intravenous infusion of β-hydroxybutyrate impaired mammary gland defense during a *Streptococcus uberis* challenge. Late lactation, multiparous Holstein cows (n = 12) were blocked by parity and milk yield and randomly assigned to either receive an intravenous infusion of 2.5 M Na-BHB to induce hyperketonemia or 2.5 M NaCl (CON) for 72 h. Infusion rates were adjusted to maintain a target concentration of 1.8 mM BHB in the BHB-infused cows. All cows were challenged with *S. uberis* in the right rear and right front quarters of the mammary gland at the start of the infusion. Milk samples collected from the front challenged quarter were used for bacterial enumeration **(A)**, identification of clinical mastitis **(B)**, and somatic cell count (SCS; **C**). Dry matter intake **(D)** and milk yield **(E)** were recorded daily. Vaginal temperature **(F)** was recorded hourly using a data logger and averaged by day. Data presented as LSM ± SE. *P ≤ 0.05, ^†^0.05 < *P* ≤ 0.10.

As expected, BHB infusion elevated circulating BHB concentrations to 1.81 ± 0.054 mM while the CON cows averaged 0.55 ± 0.053 mM BHB (**Figure 1**; *P* < 0.0001). *Streptococcus uberis* was isolated from milk samples from all cows by 24 h post-challenge, and 9 out of the 12 cows displayed clinical signs within 7 d of challenge. A significant treatment by time interaction was found for bacterial counts (interaction, *P* = 0.02), as cows infused with BHB had greater milk *S. uberis* cfu/mL (**Figure 2A**) on d 4 (*P* = 0.03), 6 (*P* < 0.01), and 7 (*P* < 0.001) post-challenge, and tended to be greater on d 3 (*P* = 0.10) and 5 (*P* = 0.07) than CON. Treatment did not influence the onset of clinical mastitis (**Figure 2B**; *P* = 0.95) nor did it affect SCS (**Figure 2C**, *P* = 0.41), however, cows infused with BHB consumed 3.1 kg/d less dry matter (**Figure 2D**; *P* = 0.02) and produced 2.6 kg/d less milk (**Figure 2E**; *P* = 0.03) than CON. Coinciding with these data, cows infused with BHB had a lesser vaginal temperature (treatment × time interaction, *P* < 0.01; **Figure 2F**) on d 3 (the final day of infusion; *P* < 0.001), but a tendency for a greater temperature on d 5 (*P* = 0.09), and a significantly greater vaginal temperature on d 6 (*P* = 0.001).

**Figure 3:**
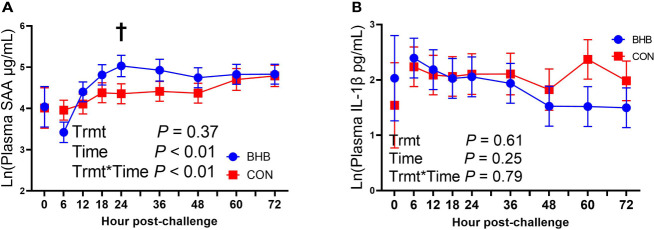
Intravenous infusion of sodium β-hydroxybutyrate did not alter systemic inflammatory mediators during a *Streptococcus uberis* challenge. Late lactation multiparous cows (n = 12) were blocked by parity and milk yield and randomly assigned to either receive an intravenous infusion of 2.5 M Na-BHB to induce hyperketonemia or 2.5 M NaCl (CON) for 72 h. Infusion rates were adjusted to maintain a target concentration of 1.8 mM BHB in the BHB-infused cows. All cows were challenged with *S. uberis* in the right rear and right front quarters of the mammary gland at the start of the infusion. Blood samples were collected every 6 to 12 h during the 72 h infusion. Serum amyloid A (SAA; **A**) and interleukin-1β (IL-1β; **B**) concentrations were determined using a bovine specific ELISA. Data presented as LSM ± SE. ^†^0.05 < *P* ≤ 0.10.

**Figure 4:**
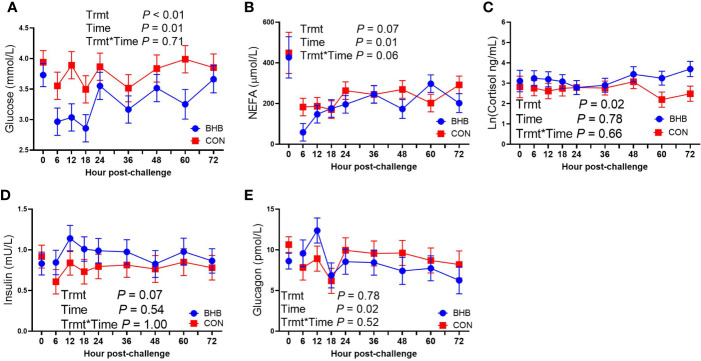
Intravenous infusion of sodium β-hydroxybutyrate altered glucose metabolism during a *Streptococcus uberis* challenge. Late lactation multiparous cows (n = 12) were blocked by parity and milk yield and randomly assigned to either receive an intravenous infusion of 2.5 M Na-BHB to induce hyperketonemia or 2.5 M NaCl (CON) for 72 h. Infusion rates were adjusted to maintain a target concentration of 1.8 mM BHB in the BHB-infused cows. All cows were challenged with *S. uberis* in the right rear and right front quarters of the mammary gland at the start of the infusion. Blood samples were collected every 6 to 12 h during the 72 h infusion. Glucose **(A)** and nonesterified fatty acid (NEFA; **B**) concentrations were determined using enzymatic colorimetric assays. Cortisol **(C)**, insulin **(D)**, and glucagon **(E)** were determined using a bovine-specific ELISA. Data presented as LSM ± SE.

Although there was a significant treatment by time interaction for SAA (interaction, *P* < 0.01; **Figure 3A**), BHB infusion did not significantly influence systemic SAA concentrations at any time point (all *P* ≥ 0.07). Similarly, BHB infusion did not affect circulating IL-1β concentrations (*P* = 0.61; **Figure 3B**) or transcript abundance in the mammary gland of either the challenged quarter (**Table 2**; all *P* ≥ 0.18) or unchallenged quarter (all *P* ≥ 0.34; data not shown). However, BHB infusion did influence metabolism (**Figure 4**). In particular, BHB cows had lesser glucose (*P* < 0.01; **Figure 4A**) and tended to have lesser NEFA concentrations (*P* = 0.07; **Figure 4B**). Coinciding with these findings, BHB infused cows had marginally greater insulin concentrations (*P* = 0.07; **Figure 4D**) in addition to greater cortisol concentrations (*P* = 0.02; **Figure 4C**) relative to CON cows. No effect of BHB was found on glucagon (*P* = 0.78; **Figure 4E**) or the insulin to glucagon molar ratio (*P* = 0.49; data not shown).

**Table 2:** Transcript abundance from mammary tissue collected from *S. uberis* right rear challenged quarter of late lactation cows that were either infused with saline (n = 6) or BHB (n = 6) to maintain a 1.8 mM concentration for 72 h.

Gene^1^	Relative mRNA abundance (Ln)	SE	Relative mRNA abundance (Ln)	SE	*P*-value
	BHB		CON		
*IL1β*	0.53	0.97	1	0.97	0.74
*IL8*	1.2	0.60	1	0.35	0.81
*IL10*	1.4	0.53	1	0.65	0.60
*TNFA*	2.0	1.1	1	1.1	0.51
*CCL5*	2.1	0.98	1	1.01	0.46
*LTF*	3.0	0.99	1	0.99	0.18
*NLRP3*	2.8	0.99	1	0.99	0.22
*GPR109A*	1.1	0.046	1	0.039	0.36

^1^IL, interleukin; TNFA, tumor necrosis factor α; CCL5, chemokine ligand 5; LTF, lactoferrin; NLRP3, NLR family pyrin domain containing 3; GPR109A, G-protein coupled receptor 109A.

Transcript abundance was normalized to *UXT* and *RPS9* and adjusted for primer efficiency using the Pfaffl (24) equation. Data were transformed using the natural logarithm.

### RAW 264.7 Cell Culture Experiment

**Figure 5:**
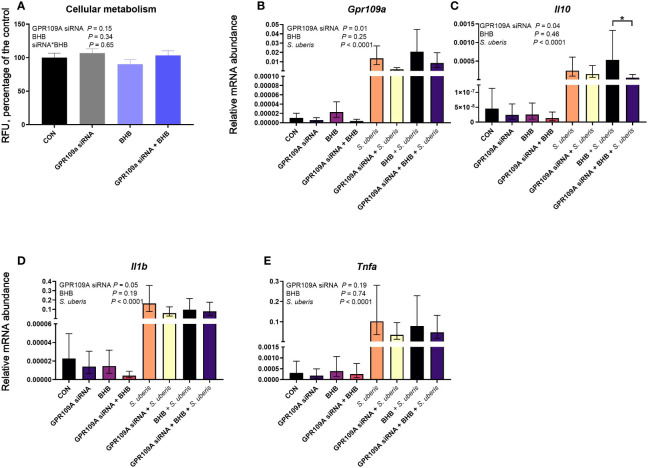
GPR109A silencing reduced *Il10* from mouse macrophages (RAW 264.7 cell line) challenged with *S. uberis*. Data were analyzed as a factorial (n = 8 or 9 replicates/treatment group for cellular metabolism and transcript abundance, respectively) with fixed effects of GPR109A siRNA, BHB treatment, and *S. uberis* challenge (transcript abundance only). Cells were either transfected with 25 nM GPR109A siRNA or with a scrambled control for 24 h. Following transfection, cells were treated with media containing either 0 or 1.8 mM BHB and were challenged with 2 × 10^7^ cfu *S. uberis* (0140J strain) or not challenged for 3 h. Cellular metabolism **(A)** was assessed by measuring fluorescent resorufin formation in relative fluorescent units (RFU) expressed as a percentage of the control. Transcript abundance of target genes (**B**, *Gp109a*; **C**, *Il10*; **D**, *Il1b*; **E**, *Tnfa*) were normalized to *B2M* using the Pfaffl (24) equation. *P ≤ 0.05.

GPR109A knockdown did not impact cell viability (*P* = 0.15; **Figure 5A**) nor did BHB treatment (*P* = 0.34). *Streptococcus uberis* challenge increased transcript abundance of *Gpr109a*, *Il10*, *Il1b*, and *Tnfa* (all *P* < 0.0001; **Figure 5**). The siRNA successfully knocked down *Gpr109a* by 75% (*P* = 0.01; **Figure 5B**). Downstream effects of GPR109A silencing were found on *Il10* (*P* = 0.04; **Figure 5C**) reducing transcript abundance by 63%. Similarly, GPR109A knockdown reduced *Il1b* (*P* = 0.05; **Figure 5D**). Treatment with BHB did not influence the abundance of any transcript measured; however, the statistical contrast revealed that GPR109A knockdown reduced *Il10* by 90% in BHB treated, *S. uberis* challenged macrophages (*P* = 0.05; **Figure 5C**) as compared to scrambled siRNA controls that were also treated with BHB and challenged with *S. uberis*.

## Discussion

Advancing our understanding of how metabolites such as BHB can influence immune function is critical to improving postpartum dairy cattle health. Decades of dairy cattle research have associated elevated BHB concentrations with disease incidence in postpartum dairy cows (1, 4, 5) as well as mastitis severity (27). Similarly, other studies have associated body weight loss in early lactation cows with clinical mastitis incidence (28), elevated SCS (29), and new subclinical intramammary infection (30). As such, feed restriction models have been used during either lipopolysaccharide (LPS; 31, 32) or *S. uberis* (33) intramammary challenges to provide further insight into these associations, but in general, these studies found only a minor impact of negative energy balance on clinical disease outcomes. Because feed restriction induces numerous physiological changes, the objective of the present study was to elucidate the direct role of hyperketonemia during a *S. uberis* intramammary challenge. Here, we build upon these past associations and demonstrate a causative role of hyperketonemia in the pathogenesis of *S. uberis* intramammary infection.

### Effects of BHB on Immune Defenses

A key objective to our study was to determine the effect of BHB on mammary gland immune defenses during a bacterial challenge. BHB infusion resulted in greater milk *S. uberis* cfu relative to CON cows, and by 7 days post-challenge, this difference had grown to approximately 6 log-fold. Our data corroborate the findings from past *in vitro* studies which showed that BHB impaired bacterial killing abilities of immune cells (8, 11, 34). However, we did not find an effect of BHB on SCS suggesting no profound impact on immune cell migration into the mammary gland. These results are unlike those of Zarrin et al. (35), where BHB infusion in late lactation cows reduced SCS during an intramammary LPS challenge. We speculate that this is likely due to differences in challenge models. Specifically, LPS would stimulate a rapid immune response within a few hours following challenge, whereas a live *S. uberis* challenge would slowly progress over the course of days, as seen in the present study as well as past studies (21, 36). Potentially, a longer BHB infusion time was needed to reveal differences in diapedesis when using a *S. uberis* challenge model. Furthermore, Zarrin et al. (35) demonstrated that BHB infusion increased *IL10*, an immunosuppressive cytokine, and we have produced similar results from *S. uberis* challenged macrophages treated with BHB (26). In the present study, we did not find any differences in transcript abundance of inflammation-related genes. We conducted our mammary biopsy at 72 h post-challenge to coincide with the end of the infusion period. However, at this time point, we were just beginning to see subtle differences in *S. uberis* cfu between treatment groups (statistical trend identified on d 3). Possibly, conducting the mammary biopsy 6 to 7 days post-challenge when larger, significant differences in bacterial counts were found would have yielded more interesting results.

The present study revealed that BHB infusion delayed the febrile response to *S. uberis* as compared to CON cows. In particular, the body temperature of CON cows peaked 48 to 72 h following challenge, whereas BHB cows were unable to mount a febrile response until after the infusion period was completed (d 5 following challenge). Although no effect of BHB was found on systemic inflammatory markers such as SAA or IL-1β, BHB infused cows had greater plasma cortisol concentrations. While the treatment by time interaction was not significant, the divergence in cortisol concentrations between treatment groups mostly occurred at 60 and 72 h following challenge, coinciding with the results for body temperature. Cortisol is a regulator for inflammatory responses, particularly known to impair innate immune responses (37), although it should be noted that this effect in humans is dependent on both concentration and time relative to the inflammatory insult (38). Nevertheless, elevated cortisol concentrations, blunted febrile responses, and sizable differences in *S. uberis* cfu from BHB infusion would suggest profound impairment of the immune system.

### Effects of BHB on DMI and Milk Production

Cows infused with BHB consumed less DM and produced less milk than CON cows. These are likely consequences of a more severe *S. uberis* intramammary infection, and not a direct result of hyperketonemia, as past continuously infused BHB studies did not impact feed intake or milk production in dairy cows (19). While clinical ketosis is associated with reductions in DMI and milk production in postpartum dairy cows (39), it is also associated with inflammation (40). Indeed, studies inducing pro-inflammatory states in early lactation cows have shown reductions in milk production and feed intake (41, 42). Hence, while it may be appealing to conclude a direct effect of hyperketonemia on appetite regulation and milk production in the present study, we speculate that these differences are simply a downstream effect of impaired immune function and subsequently a more severe intramammary *S. uberis* infection. With that said, studies using a intracerebroventricular infusion of a BHB bolus did reduce feed intake in dairy cows (43), and therefore additional research is needed to clarify the role of BHB on appetite regulation.

### Effects of BHB on Glucose Homeostasis and Its Interaction with Immune Function

Cows with ketosis commonly have depressed blood glucose concentrations (44). Our study as well as other BHB infusion studies conducted in ruminants have consistently found a decline in glucose due to hyperketonemia (19, 45, 46). Glucose is a critical energy source for activated immune cells (47) and therefore reductions in this substrate could alter immune responses (10, 48) which could possibly explain some of the differences found in *S. uberis* cfu. With that said, the difference in *S. uberis* cfu was mostly noted after infusion, and it seems unlikely that glucose levels would have remained reduced from BHB treatment for days following infusion (45), casting some doubt on this theory. Nevertheless, BHB cows in the present study also had greater cortisol concentrations, a steroid known to stimulate gluconeogenesis. As such, these data could represent a homeostatic mechanism used to restore glucose concentrations (49). It should be noted however that glucose levels in BHB cows remained lesser than CON throughout the infusion suggesting that the effect of BHB on cortisol may not be substantial enough to restore glucose concentrations. Still, a detrimental side effect of this homeostatic mechanism would be impairment of the immune system from elevated cortisol levels. Thus, our data might underscore the need to improve metabolic adaptations in early lactation cows to enhance resistance toward infectious disease agents.

While the effect of BHB infusion on glucose concentrations is consistent across stage of lactation and gestation in dairy cows (19, 45), the mechanism behind this remains unclear and likely depends on the physiological state of the cow. Specifically, BHB infusion increased insulin concentrations in prepartum cows, however, had no impact on insulin in postpartum cows (45). In mid to late lactation cows, BHB infusion had no impact on insulin concentrations, but reduced glucagon concentrations (19, 50). Our study failed to provide any clarity on the mechanism behind the reductions in blood glucose concentrations, as only a statistical trend for greater insulin levels were found in BHB infused cows. Nevertheless, it should be noted that a BHB infusion into cows in positive energy balance, such as the late lactation cows used in the present study, would likely impact metabolism differently than an infusion into early lactation cows experiencing negative energy balance. In particular, BHB can be used as an energy source, and therefore to some degree it would substitute the energy demand for glucose (19, 50).

### Potential Mechanisms

Investigators have found numerous ways BHB can alter cellular responses (12), one of which is through activation of the G-protein-coupled receptor, GPR109A. Because past research found that BHB induced an *Il10* response (13, 26, 35), we next sought to determine if this effect was mediated through GPR109A signaling using mouse macrophages challenged with *S. uberis*. Unlike our previous work (26), the present study did not find a significant effect of BHB treatment on *Il10*, however, transcript abundance of this cytokine was numerically greater due to BHB treatment. Moreover, GPR109A knockdown significantly reduced *Il10*, in agreement with Chen et al. (13), suggesting that BHB effects on *Il10* are likely mediated through GPR109A. Future studies using bovine macrophages are needed to determine if this effect is conserved across species. In particular, investigating the role of GPR109A on reduced phagocytosis seen in BHB-treated bovine milk macrophages (11) may be informative.

One intriguing aspect of our data is the profound effect of BHB infusion on bacterial counts following the infusion, even though only minor effects were found during infusion. Our data might suggest that BHB has a carry-over effect on immune function. Specifically, BHB is an inhibitor of histone deacetylase (12), which is an enzyme that removes acetyl groups from histones to exert long-term effects on the regulation of gene expression. It seems plausible then that BHB could impair immune activation and recognition of pathogens even after ketone levels return to normal concentrations. As such, impairment of the sentinel function of the immune system and mammary epithelium may be related to epigenetic regulation of inflammatory-related genes. Therefore, future *in vivo* studies should consider this possibility as BHB-induced epigenetic alterations could contribute to mastitis susceptibility and severity.

## Conclusion

In conclusion, elevated ketone concentrations have long been associated with infectious disease incidence in dairy cows. Our study is the first to demonstrate a direct role of BHB in the pathogenesis of *S. uberis* mastitis. Hyperketonemia impaired immune defenses noted by greater *S. uberis* cfu in milk and impaired febrile responses following the challenge and subsequently, BHB infused cows consumed less DM and produced less milk. Potentially, impaired immune responses were the result of altered metabolism as cows infused with BHB had lesser glucose and greater cortisol concentrations. The mechanism behind these effects still requires further investigation.

## Data Availability Statement

The original contributions presented in the study are included in the article/supplementary material. Further inquiries can be directed to the corresponding author.

## Ethics Statement

The animal study was reviewed and approved by Kansas State University Institutional Animal Care and Use Committee.

## Author Contributions

TS conceptualized the study, conducted the experiments, analyzed the data, and prepared the manuscript. BB supervised the experiments and provided edits for the manuscript. LM provided support for experimental procedures involving molecular biology techniques. All authors contributed to the article and approved the submitted version.

## Funding

This work and the primary author are financially supported by a USDA postdoctoral fellowship (2019-67012-29665).

## Conflict of Interest

The authors declare that the research was conducted in the absence of any commercial or financial relationships that could be construed as a potential conflict of interest.
